# Medicinal Plants Used to Treat Skin Diseases and for Cosmetic Purposes in Norway

**DOI:** 10.3390/plants13192821

**Published:** 2024-10-09

**Authors:** AmalaChukwu M. Ijeabuonwu, Jurga Bernatoniene, Zivile Pranskuniene

**Affiliations:** 1 Department of Drug Technology and Social Pharmacy, Lithuanian University of Health Sciences, LT-50162 Kaunas, Lithuania; amalachukwu.mary.ijeabuonwu@stud.lsmu.lt (A.M.I.); jurga.bernatoniene@lsmu.lt (J.B.); 2 Institute of Pharmaceutical Technologies, Lithuanian University of Health Sciences, LT-50162 Kaunas, Lithuania

**Keywords:** Norway, ethnobotany, skin diseases, cosmetics

## Abstract

Skin diseases in Norway represents an important area of study due to their incidence and prevalence, yet ethnobotanical research in this context remains scarce. The aim of this study was to evaluate the knowledge of Norwegian traditional medicine regarding the treatment of skin diseases and cosmetic purposes through a comparative analysis with EMA monographs. Participants were surveyed using semi-structured interviews. The two samples comprised 22 individuals from Bodø and 26 individuals from other Norwegian communities, all of whom use medicinal plants for the treatment of skin diseases and cosmetic purposes. The indications for skin diseases identified in the study were compared with the European Union herbal monographs published by the European Medicines Agency. Fieldwork was conducted between June 2022 and September 2023, during which 42 plant species from 22 families were recorded. More than half of these plant species (65.38%) were used without European Medicines Agency-approved medical indications. From a public safety perspective, ensuring the safety of self-treatment for skin diseases is a critical research focus for future ethnobotanical studies.

## 1. Introduction

Among the conditions that most commonly affect human health, skin diseases are particularly significant, as they can severely impair individual well-being. This issue is a major global concern. Skin diseases are the most frequent reason for consultation in general practice [1], and curing mild conditions is attempted with herbal homemade preparations. As the prevalence of skin diseases continues to rise, so does the interest in herbal medicine. Herbal remedies are believed to have fewer side effects and disadvantages compared to modern pharmaceuticals [2,3]. 

Despite the widespread availability of professional medical care in Europe, residents continue to prepare homemade products for the treatment of skin conditions and for cosmetic purposes [4,5,6]. Analyzing skin diseases in Norway is particularly important due to the incidence and prevalence of these conditions, yet ethnobotanical research remains scarce [7]. In Norway, diverse indigenous and immigrant groups bring their own traditional practices, further complicating the study of traditional medicine. The leading ethnobotanist in Norway, Ove Arbo Høeg (1898–1993), dedicated over 50 years to documenting the use of plants in Norway. In his 1974 publication “Planter og tradisjon,” Høeg noted that while some knowledge had been lost, many plant use traditions persisted even after 200 years [8,9].

Regarding the collection of modern ethnobotanical knowledge in Norway, research in the field expresses different opinions. On one hand, it is in some cases claimed that there is little or nothing left to collect, and on the other hand, it is also suggested that fieldwork is still possible in many northern European countries including Norway as well [10]. Existing studies show that traditional medicine is still prevalent in modern Norway, with the use of medicinal herbs and natural remedies on the rise [10,11,12]. Ethnobotanical studies regarding skin diseases and cosmetics are usually researched in the countries where primary medical care is difficult to access [2,3,13,14,15,16,17,18,19,20,21]. Although nearly all European ethnomedicine articles mention skin diseases and wound healing, some studies have also focused separately on skin diseases, wounds, and folk cosmetics [4,5,6,22,23]. We have highlighted only a few examples from the extensive research on ethnomedicine, specifically focused on the treatment of skin diseases and cosmetic applications.

Globally, there is a growing interest in local traditional knowledge, which has led to the documentation and scientific analysis of traditional recipes. Ethnopharmacological studies often validate the traditional uses of medicinal plants; however, in some cases, the indications differ significantly. It is valuable to assess the extent to which the information collected during ethnobotanical research aligns with data derived from scientific studies. The European Medicines Agency (EMA) has published monographs for certain herbal materials, offering safety and efficacy data based on rigorous scientific evaluations. However, many commonly used plants either lack EMA monographs or are utilized for purposes not covered by these monographs. The aim of this study was to evaluate the knowledge of the Norwegian traditional medicine regarding the treatment of skin diseases and cosmetic purposes through a comparative analysis with EMA monographs. 

## 2. Results and Discussion

### 2.1. Respondents from Norwegian Communities

A total of 48 respondents were surveyed, comprising 22 individuals from Bodø (Sample P1) and 26 individuals found online from 7 out of 10 counties (Sample P2). In the P1 group, there were 20 women and 2 men, whereas Sample P2 included 25 women and 1 man. 

Jost et al. [24] studied the ethnobotanical plants used for cosmetic purposes in French Polynesia, selecting 28 respondents based on their extensive knowledge of traditional plant use and their production of homemade cosmetic preparations. Of these respondents, 82% (23) were women and 18% (5) were men. These findings align with observations in Lithuania [4], where ethnobotanical knowledge and tradition are more prevalent among women than men. The authors noted that the female-to-male ratio was unintentional. Since the study focuses on the cosmetic use of herbal materials, a domain of greater concern to women, it is logical that women would predominantly prepare and use such herbal recipes. In contrast, in Morocco [2], a study found that 95.4% of herbalists were male. The authors attribute this to Moroccan cultural norms that discourage women from engaging in this type of work, making it a predominantly male domain. Thus, the women-to-men ratio highly depends on cultural and in-country practices and beliefs. 

Most respondents from Sample P1 had a higher education (62.50%), with the lowest level of education being upper secondary school (37.50%). The occupations of individuals surveyed in P1 varied widely. Among those who responded positively, six were retired (one of whom is a botanist), three were shop assistants, six were in healthcare (including one physician, one nurse, one certified nursing assistant, one social worker, one dentist, and one uncertified nursing assistant), one was a salesman, one was an artist, two were kindergarten teachers, one was a historian, one was an auditor, and one was a specialist in design operations.

**Figure 1:**
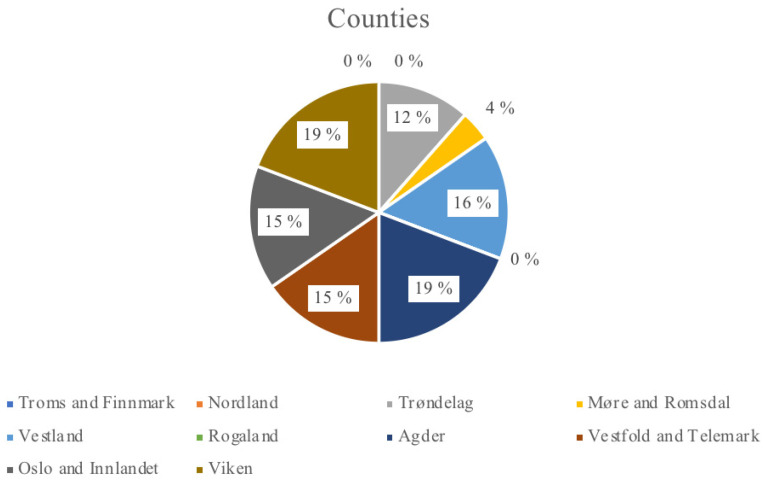
Counties represented by the respondents of P2.

In the P2 group, all participants either produced their own or used herbal remedies for the treatment of skin diseases and/or cosmetic purposes, with some also selling their products. Most respondents were from Agder and Viken, each representing 19% of the total (Figure 1). The age range of respondents in P1 group was 18–103 years, in the P2 group it was 31–80 years. In the P1 group, the majority of respondents were from the 20–29 age group, while in the P2 group, most respondents belonged to the 30–39 age group. 

**Figure 2:**
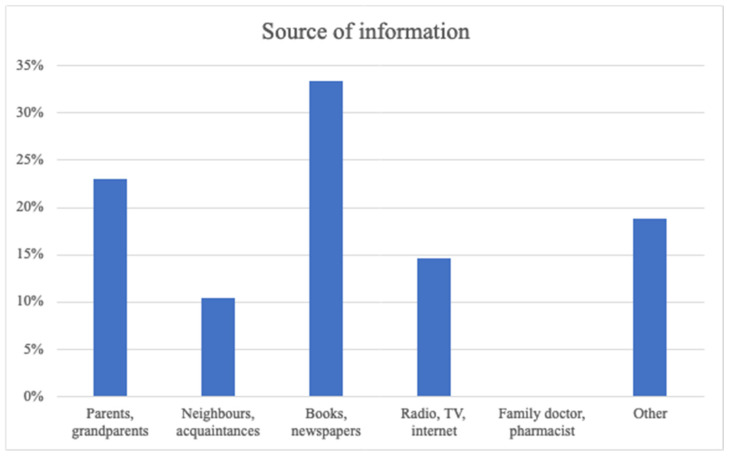
The main sources of information for the individuals.

Similar to the P1 sample, most of respondents did not seek consultation from pharmacists or physicians regarding the use of medicinal herbs, aligning with the findings of Djuv et al. [25]. The reasons for not consulting healthcare professionals varied. Some respondents perceived that “healthcare professionals, such as pharmacists and doctors, lacked knowledge about herbal medicine.” One individual even suggested that “doctors and pharmacists are opposed to it.” Another respondent mentioned “a sense of failure by the healthcare system.” The main sources of information regarding traditional medicine to treat skin diseases and for cosmetic purposes were books, newspapers and relatives (Figure 2).

Additionally, 73.08% of the P2 sample reported that people approached them for help in finding herbal raw materials, and 96.15% stated that they passed on their knowledge of herbal treatments to others. 

### 2.2. Raw Materials for Skin Diseases and Cosmetic Purposes 

All results from the study are systematized in Table S1, “Medicinal plants used to treat skin diseases and for cosmetic purposes in Norway” (Supplementary Materials Table S1).

In the P1 sample, nine different plant species from eight families were mentioned. Although the P2 sample was smaller, it included a greater variety of plants, with a total of 40 plant species from 20 families.

In the P1 group, the most frequently cited family was Lamiaceae, whereas in P2 group, it was Asteraceae, followed by Lamiaceae. These findings are consistent with studies conducted in Turkey [26], Italy [5], Lithuania [4], and regions in and around Eastern Europe [27].

**Figure 3:**
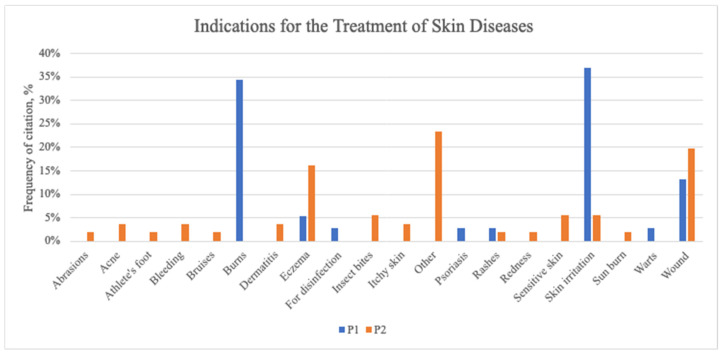
Combined results of both communities of indications for the treatment of skin diseases.

The comparison of the results indicates a greater variety of skin disorders treated in the P2 sample compared to the P1 sample (Figure 3).

In the P1 sample, the primary indication was skin irritation, followed by skin burns. Conversely, in the P2 sample, wounds were the most cited indication, followed by eczema. In the Lithuanian study conducted during COVID-19, the main indication was also wounds (39%) and this was the main indication from the category of skin diseases in other studies as well [4,20]. Tsioutsiou et al. [27] observed that wounds and burns were the primary skin-related problems treated with medicinal plants. 

The most frequently cited plants for the treatment of skin diseases in our study were *Calendula officinalis* L. (17.32%), followed by *Plantago major* L. (9.45%) and *Matricaria recutita* L. (6.30%). *Matricaria recutita* L. and *Calendula officinalis* L are the most known and commonly used medicinal plants for skin diseases [28], and studies have even focused on the potential anticancer actions of these extracts [29].

The leaves of *Plantago major* L. and *Achillea millefolium* L. were often cited for wound treatment. Some respondents applied the leaves directly to the skin, while others added them to ointments with other herbs or made ointments using a single plant. 

*Plantago major* L. leaves and *Achillea millefolium* L. have been used to treat trauma and wounds in several parts in Europe [4,22]. Traditional use of these plants in healing is confirmed by *in vitro* and/or *in vivo* studies [22]. Additionally, some individuals create soaps or shampoos with these plants. According to respondents, *Trifolium pratense* L. flowers can be used in ointments to treat psoriasis or as an oil in facial scrubs. In addition to other traditional applications, this plant is known to be used in European cultures for the treatment of eczema and psoriasis [30]. Respondents in our study also mentioned using *Allium cepa* L. for wart removal. In their study of medicinal plants used to treat dermatological issues in the South Balkan and East Mediterranean region, Tsioutsiou et al. [27] found that *Allium cepa* L. was among the most frequently cited plant species for treating skin-related conditions. 

In Population P2, the category of “other” had the most citations (22.81%). This category includes a range of conditions such as the improvement of liver function, relaxation, chest tears, stress balance, rheumatoid arthritis, sore muscles, pH imbalance, chronic skin inflammation, tender varicose veins, hemorrhoids, rheumatism, lymphatic system support, improved circulation, and nosebleeds (Figure 3). 

**Figure 4:**
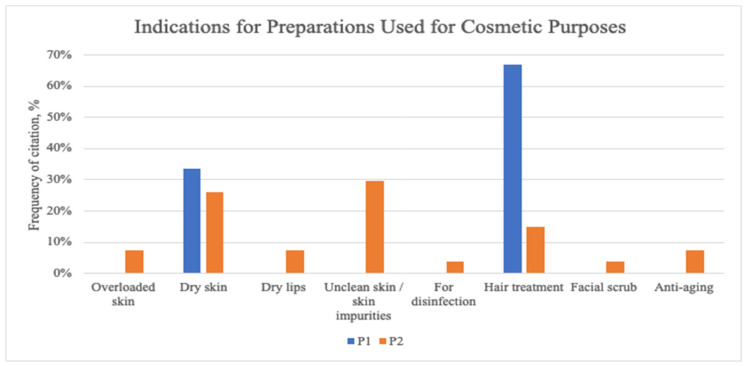
Combined results from both communities of indications for herbal materials used for cosmetic purposes.

In P1, only two cosmetic uses of plants were mentioned, which were hair treatment (66.67%) and dry skin (33.33%). In P2, the most cited cosmetic use of plants was to treat skin impurities and dry skin (29.63%) (Figure 4). 

**Figure 5:**
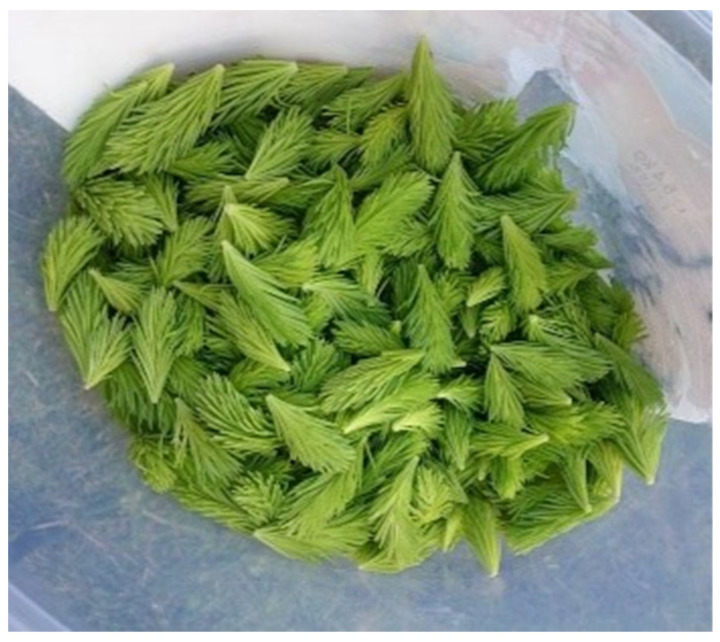
*Picea abies.* Preparation for oil production. Informant from Population P1, aged 65 years, occupation: agricultural worker. Figure were taken on 27 May 2022 in Notodden.

**Figure 6:**
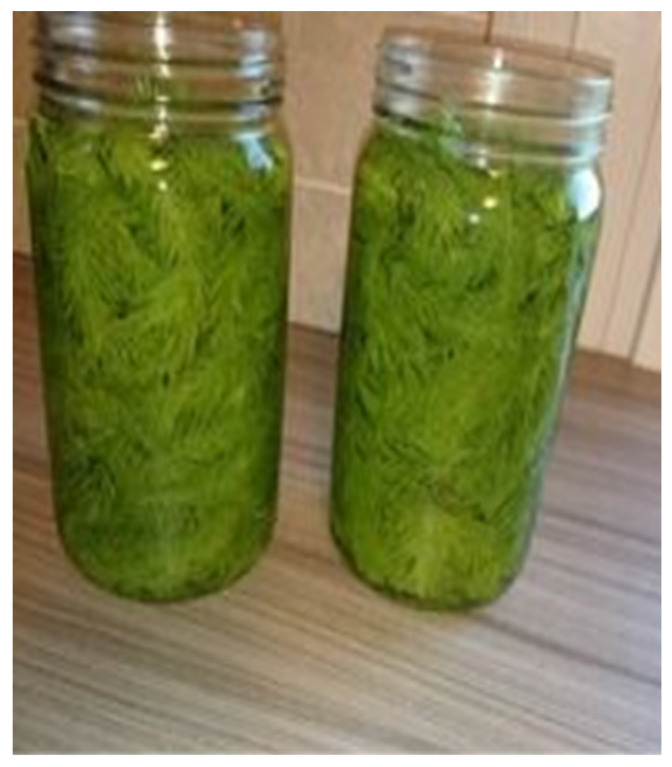
*Picea abies*. Preparation for oil production. Informant from Population P1, aged 65 years, occupation: agricultural worker. Figure were taken on 27 May 2022 in Notodden.

Skin and hair hydration were the main indications for cosmetic purposes in the ethnopharmacological study dedicated to the category of skin diseases and cosmetics in Lithuania [4]. To solve these cosmetic problems, handmade oils and soaps were produced. In practical applications, the leaves of yarrow and broadleaf plantain can be rinsed, dried, and placed in a jar of oil for approximately two weeks to produce an herbal oil, which can be used in the production of ointments. The same process applies to red clover, with the flowers being the utilized parts. Shoots of the Norway spruce are used to make soaps. To make soaps, one must first produce oil through oil extraction (Figure 5 and Figure 6).

In the P1 sample, the most popular method of preparation was juicing, accounting for 25%, followed by oil extraction and decoction, each at 16.67%. The juice of *Aloe vera* Mill. is commonly used to treat burned or irritated skin, as well as wounds. While the leaf can be applied directly to the skin, users reported that the juice has the primary therapeutic effect. One respondent mentioned rolling cabbage leaves to soften them before applying them to her skin for wound healing. 

Some plants were used internally as food or drink for their therapeutic effect on skin diseases. Examples of internal administration methods include eating the plant as food or drinking it as a tea or in a smoothie.

In the P2 sample, the most cited method of preparation was decoction (28.36%), followed by oil extraction (23.88%). Some informants mentioned extraction as a preparation method without further specification. Some plants were used internally, either as food or drink, for their therapeutic effects on skin diseases. Internal administration methods also included eating the plant, drinking it as a tea, or incorporating it into a smoothie.

**Figure 7:**
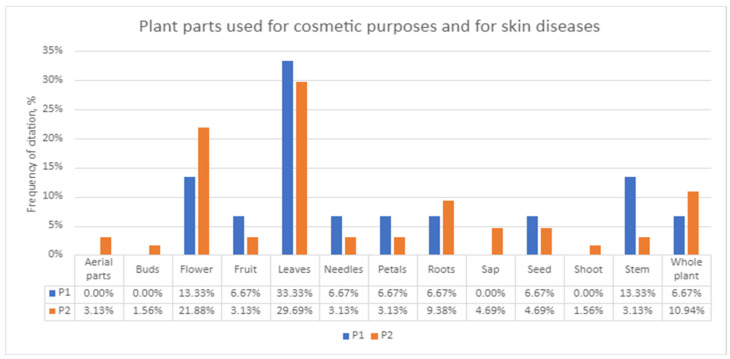
Plant parts cited by both populations.

The most cited plant parts used were leaves (30.38%) and flowers (20.25%) (Figure 7).

In the P1 sample, only two informants emphasized the importance of natural conditions for the collection of medicinal plant raw materials. In contrast, in the P2 sample, most of respondents (96.15%) highlighted the significance of natural conditions for herb collection. Seasonality and weather conditions were the most frequently cited factors important for the collection of herbs.

**Figure 8:**
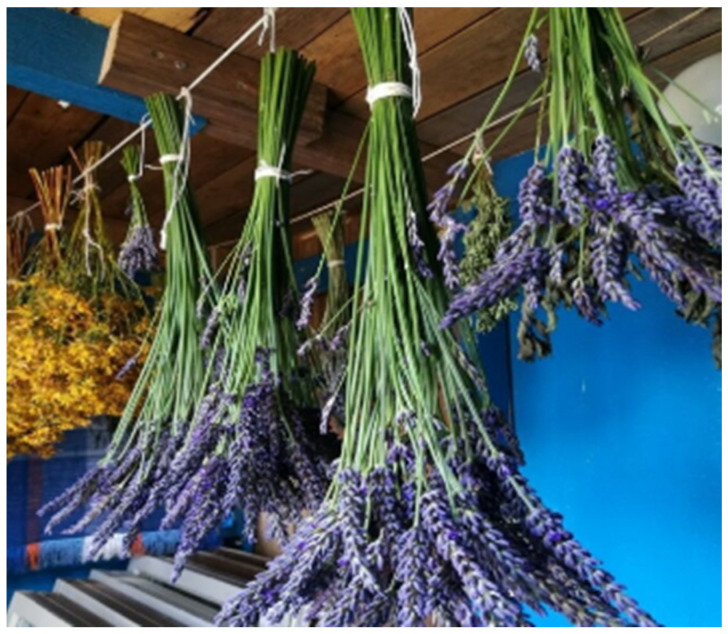
Drying of medicinal plant raw materials: *Lavandula spica*. Photos were captured in Horten, on 4 July 2022.

**Figure 9:**
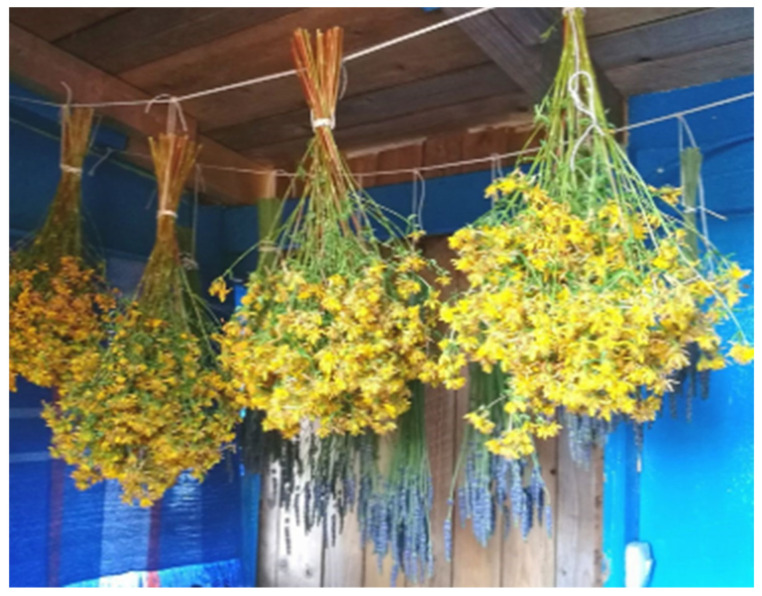
Drying of medicinal plant raw materials: *Hypericum perforatum*. Photos were captured in Horten, on 4 July 2022.

Regarding the storage of herbal raw materials, the most cited condition was storing in the dark (26.47%), followed by storing in jars or glass containers (16.18%) and in dry conditions (14.71%). Two individuals mentioned that herbal raw materials should be hung in a cold and dry place (Figure 8 and Figure 9).

### 2.3. Comparison of Medicinal Plant Raw Materials Used in Norwegian Communities with Recommendations in EMA Monographs

The study recorded 42 plant species used for skin diseases and cosmetics, belonging to 22 families. Of these, 38.09% (16 species) are not included in the official herbal monographs of the European Medicines Agency (EMA). Among the species that are included, 34.62% (9 species) were used with EMA-approved medical indications for skin diseases.

In the P1 sample, nine plant species used for skin diseases and cosmetics were identified, belonging to eight families. Of these, three species (33.33%) were used in accordance with their EMA herbal monographs. Three species (33.33%) were not included in the official EMA herbal monographs, and the remaining three species (33.33%) were included in the EMA monographs but were used for purposes other than their ascribed indications.

In the P2 sample, 40 plant species used for skin diseases and cosmetics were identified, belonging to 20 families. Of these, 36.59% were used without an official EMA herbal monograph, and only 24.39% of the taxa were used in accordance with their EMA-approved indications. In the Lithuanian study conducted during the COVID-19 pandemic, 43 of 67 mentioned plant species were not included in the EMA monographs and only 14 species (21%) of all included species were used with EMA-approved medical indications for the treatment of skin diseases. Other medicinal plants were used without EMA-approved medical indications and were based solely on folk knowledge and experience in medicine [4]. A comprehensive study from Latvian archival sources highlighted that one of the most common health conditions were skin disorders. Analysis of EMA monographs showed that only 59 out of 211 taxa mentioned in this study are included in the official European Union herbal monographs [31]. 

According to the EMA, one of the most cited medicinal plants, *Calendula officinalis* L., can be used for the symptomatic treatment of minor skin inflammations, such as sunburn, and to treat minor wounds. Informants reported using *Calendula officinalis* L. for treating dermatitis, eczema, athlete’s foot, acne, minor wounds, reducing rashes, softening hands, and as a lip balm. *Plantago major* L., which also lacks an EMA monograph, is used for wound healing.

Medicinal plants such as *Eucalyptus globulus* Labill., *Salvia Rosmarinus* L., *Salvia officinalis* L., *Lavandula Spica* L., *Hyssopus officinalis* L., and *Aloe vera* Mill. do not grow in Norway and thus are not harvested, but must be bought from pharmacies, shops, or online. According to the results of our study, many of the respondents (85%) did not seek consultation from a pharmacist or a physician regarding the use of medicinal herbs, they only started using medicinal plants. As an example, one of the most popular plants used for skin diseases and cosmetics, used for wound healing and dry skin according to our study (*Aloe vera* Mill.), does not have EMA monographs for these indications. The same is true for both *Lavandula spica* L. and *Salvia officinalis* L.; they have no cosmetic indications but are used to treat wrinkly skin (Supplementary Materials Table S1). *Salvia rosmarinus* L. is used for hair, mild psoriasis, and eczema on the scalp; however, according to EMA monographs, it is approved only for symptomatic relief of dyspepsia and mild spasmodic disorders of the gastrointestinal tract, for the relief of minor muscular and articular pain, and in minor peripheral circulatory disorders. *Eucalyptus globulus* Labill. is approved for the relief of coughing associated with a cold and the symptomatic relief of localized muscle pain, and according to our study it is used to relieve the symptoms of dry skin (Supplementary Materials Table S1). 

According to our results, most individuals do not consult a primary care specialist regarding the use of herbal preparations and only one in three medicinal plants is used according to EMA recommendations. For these reasons, it is crucial to identify the sources of information that people rely on. Our study found that the primary sources of information included books, newspapers, and mass media, as well as knowledge passed down from parents and relatives (Figure 2). Other sources of information were mentioned as well. A few examples can be described: one respondent had studied herbal medicine, another respondent is an ethnobotanist, one informant learned about the medicinal use of plants in a course for agricultural workers, and a few respondents received training from professionals. No consultations with a healthcare professional were provided. The reasons why people did not consult a healthcare professional varied. A few of the respondents mentioned that they perceived that healthcare professionals such as pharmacists and doctors lacked knowledge surrounding the subject, and one person even said that doctors and pharmacists are against herbal medicine. One individual mentioned that she felt that “the healthcare system had failed her”.

### 2.4. The Importance, Weaknesses, and Reliability of the Results

The study results present several limitations. The positive findings from the P1 sample were insufficient to draw definitive conclusions about ethnobotanical trends in Bodø. Some informants mentioned using medicinal plants but could not recall the names, leading to potential inaccuracies in the identification of plant species and families. Only two informants harvested their herbal raw materials, except for those who used aloe vera leaves or broadleaf plantain directly on their skin.

As noted in the literature review, Norway hosts a diverse array of ethnic groups, including both indigenous populations and immigrants. A notable limitation of this study is the lack of ethnic differentiation, resulting in a P1 sample comprising various ethnicities. Seven respondents were not ethnically Norwegian, and three were of Sami descent. This diversity might skew the results, as some responses may reflect non-Norwegian cultural practices rather than traditional Norwegian ones. However, this diversity also mirrors modern Norwegian culture, characterized by a multicultural population. Two Sami respondents mentioned specific natural substances: chaga and common juniper. One of these respondents is originally from Finnmark. These findings align with Kristoffersen et al. [32], who found that the use of traditional medicine in Norway is associated with the Sami ethnicity and originating from Finnmark.

Two distinct methods were employed in this study. The first method involved face-to-face interviews (P1 group), while the second method involved online interactions (P2 group). The selection criteria for informants were influenced by the COVID-19 pandemic, leading to the use of digital methods to recruit the sample of the P2 group from across Norway. However, challenges arose, as some participants refused to share recipes, while others redirected us to their websites. Our study reveals that the individuals in P1 group offer greater insight into the general population’s knowledge of medicinal plants, whereas the individuals in the P2 group primarily reflect the expertise of herbal medicine practitioners. These differences underscore that, in ethnobotanical studies, the depth of knowledge is more critical than the number of informants, as evidenced by the comparison of knowledge between these two groups. Notably, the level of knowledge regarding skin diseases and cosmetics obtained from the P1 group alone would not have been as comprehensive as that acquired from the digitally recruited and interviewed P2 group.

## 3. Materials and Methods

### 3.1. Data Collection

The fieldwork took place between June 2022 and September 2023 in Bodø in the Salten district of Norway and online. Throughout the time of this study, 48 respondents were surveyed, of which 22 individuals were from the Bodø region and 26 persons were found online. Regarding permission in Norway, De Nasjonale Forskningsetiske komiteene (National Research Ethics Committees) as well as the Regional Committee for Medical and Healthcare Research Ethics were consulted. According to the National Research Ethics Committees, a study such as this one does not require permission to be conducted. 

Local inhabitants of Bodø as well as individuals from different parts of the country were interviewed. The “snowball” method was employed to seek respondents. This study was conducted using a semi-structured interview method with a ready-made questionnaire. The questionnaire had 17 questions (Supplementary Materials File S1). The study was conducted following the guidelines of the International Society of Ethnobiology Code of Ethics [33]. The initial phase focused on gathering demographic data through primary questions and assessing the sources of ethnobotanical information. Additionally, it evaluated how many respondents consulted healthcare professionals as qualified consultants regarding the usage of medicinal plants for skin diseases and cosmetics. Emphasis was placed on identifying the sources of ethnobotanical knowledge reported by the respondents.

The objective of the second phase was to collect comprehensive information about homemade herbal preparations used for skin diseases and cosmetic purposes. This included details on their preparation methods, indications for use, dosages, duration of use, and storage conditions. Data were collected through free-form interviews. With the respondents’ permission, the interviews were voice or video recorded and notes were taken and encoded. 

The desired outcome was to find people with an abundance of knowledge about the topic. Attempts were made to find respondents among the elderly in Bodø, since they would be more likely to know more about the subject. According to Teixidor-Toneu et al. [8], the knowledge about the traditional use and medicinal properties of plants in Norway has decreased over the centuries, and thus it is to be expected that the older generation would have a richer knowledge surrounding the subject than the younger generation. The plan was to speak to the elderly in familiar neighborhoods as well as with older acquaintances and to have them provide referrals to other people. Additionally, with the anticipation of increasing the chances of finding more people who have relevant ethnobotanical knowledge, there was no discrimination regarding age; hence, adults of all age ranges were spoken to.

The COVID-19 pandemic, as highlighted by ethnobotanists from various parts of the world, necessitated adaptation to the circumstances, leading to the adoption of digital methods for information collection and surveys [34,35]. The second population was found using the internet. Specifically, the Google search engine was employed. Keywords such as “ringblomst” (marigold flower) or “groblad” (broadleaf plantain) or “einer” (juniper) were combined with words such as “salve” (ointment) or “krem” (cream) or “såpe” (soap). Occasionally, the word “hjemmelaget” (homemade) was added. The choice of plants was initially based on the trends that were observed in the responses from the Bodø respondents. With this method, more people were found and selected based on their perceived abundant ethnobotanical knowledge. 

Efforts were made to gather as much information as possible about products of herbal origin, to identify medicinal raw materials used for medicinal and cosmetic purposes, their preparation methods, indications for use, doses, duration of use, and storage conditions. The botanical nomenclature followed World Flora Online plant list [36]. The persons were asked to recognize all the used species by pictures, using encyclopedia, atlases of traditional flora of Norway [37,38] and *in vivo* plants. Some plants were identified based on their vernacular names and a full description provided by the interviewee.

The data collected in this study were compared to the indications approved by the EMA for each medicinal plant [39]. A European Union (EU) herbal monograph, previously known as a Community Herbal Monograph, provides a scientific assessment of the safety and efficacy of herbal substances and their preparations intended for medicinal use. These monographs evaluate all available information, including non-clinical and clinical data, as well as documented long-standing use and experience within the EU. The purpose of this comparison was to determine the percentage of indications identified in the study that aligned with those approved by the EMA.

### 3.2. Study Area

**Figure 10:**
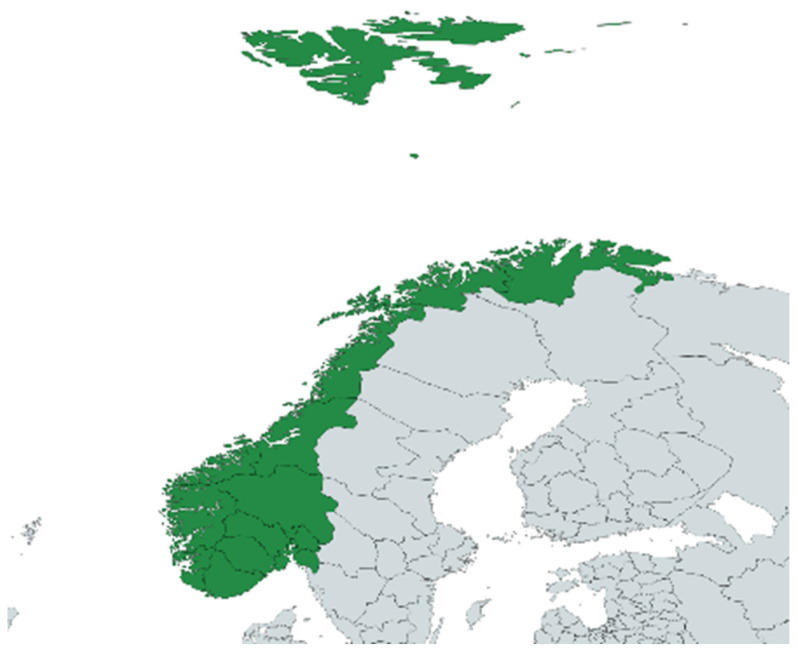
Norway on the map of Europe.

**Figure 11:**
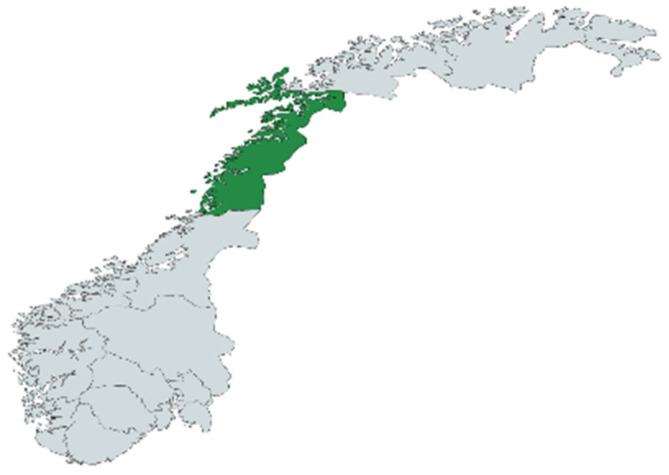
Nordland county in Norway. The county in which Bodø is the center of administration.

The study was conducted on-site in Bodø in the Salten district of Norway. Figure 10 and Figure 11 show images of Norway and of Nordland County.

Bodø municipality is a peninsula located in the northern part of Norway. It has a population of 53,712 as of 2023 [40]. The city of Bodø was established on the 20th of May in 1816, initially with the purpose of being a town for trade among the fishermen of northern Norway. The idea behind this was to reduce the dependence of the northerners on the merchants in Bergen [41].

Bodø is known for its beautiful, mountainous nature and many trails in big forests. Bodø has several lakes and rivers, and much of the city is surrounded by the ocean. Additionally, it is surrounded by fiords and pinnacles. The climate in Norway is characterized by its geographical location. The weather conditions of the country can be described as varied. The mountains have a shielding effect, thus creating a more continental climate in certain parts of the country, while other parts of the country must endure strong winds. The climate in Bodø is subarctic maritime. It is a rare kind of climate which is found mainly along the coast of Northern Europe as well as in the southern side of Alaska. On the western side of the city, there are islands and islets. Roughly one-third of the municipality is less than 60 m above the ocean. Due to the coastal climate, the city enjoys relatively cool temperatures all year round. Bodø is regularly exposed to rainfall as well as wind brought on by the Western fiord (Vestfjorden) [41].

In Norway, there are roughly 2000 plant species, and Oslo is the municipality with the richest amount of plant species, with about 796 species. There are some hundred different, red-listed plant species in the Bodø region of northern Norway, and there are some arctic plant species found there as well [42,43,44].

### 3.3. Formation of the Research Sample

In total, 176 respondents were surveyed, of which 65 individuals were from Bodø (Population P1) and 111 people (Population P2) were found online. The main difference between the two populations is that the informants of the P2 population were specifically sought out and selected because of their apparent knowledge about the use of medicinal plants, while in the Bodø population the desire was to find people with ethnobotanical knowledge. 

Initially, the topic was introduced to the individuals surveyed in the Bodø area. The aim was to use the “snowball” method to search for respondents. That is, the expectation was that as people were being surveyed, individuals would recommend other people that they knew of who had the relevant knowledge. In total, 65 people were surveyed and 66.15% (43) of them had no experience or knowledge about the traditional medicinal or cosmetic use of plants. Furthermore, out of the 65 people, only 3 people recommended 1 person each. Some of the respondents, including a retired couple (83 years old) and a retired nurse (73 years old) said when presented with the thesis, that “only the old people know about this, and we are the only old ones now” and that “it [the use of medicinal plants] is not common here.” Finally, the P1 group consisted of 22 individuals with some knowledge on medicinal plants for skin diseases and cosmetic purposes. This was the rationale for choosing two populations. Understanding that the results would not be sufficient, it was decided to use the internet to find people with extensive knowledge and experience surrounding Norwegian ethnobotany. 

With the method employed, more than 100 individuals as well as businesses (often owned by private persons) were found who produced their own herbal handmade products for skin diseases and cosmetics. Some of these were sellers of their handmade products, while others published their recipes to promote the use of natural homemade products. In total, 111 businesses and individuals were contacted via e-mail, social media, or their own personal websites. Simultaneously, each respondent was invited to do the interview over the phone or online. P1 group consisted of 26 individuals. All of them either made their own and/or used herbal remedies for the treatment of skin diseases and/or cosmetic purposes. That is, all of them make their own herbal remedies for personal use and some of them also sell their products.

## 4. Conclusions

This study represents the first comprehensive investigation into the use of medicinal plants for treating skin diseases and for cosmetic purposes in Norway. The COVID-19 pandemic has introduced new opportunities for the search of respondents and organization of the study. Our findings highlight the importance of monitoring the appropriate use of ethnobotanical materials in urban areas with widespread internet access. The availability of ethnocosmeceuticals and homemade herbal preparations online raises critical scientific concerns regarding their efficacy and safety. With the increasing demand for natural cosmetics and treatments for skin conditions, individuals often turn to social media for information and may use herbal products that lack approval from the European Medicines Agency (EMA). According to our research, over half of the plant species studied (65.38%) were utilized without EMA-approved medical indications. From a public health perspective, the safety and efficacy of self-treatment for skin diseases can be a research question for future ethnobotanical research.

## Data Availability

The original contributions presented in the study are included in the article/Supplementary Material, further inquiries can be directed to the corresponding author.

## Supplementary Materials

The following supporting information can be downloaded at https://www.mdpi.com/article/10.3390/plants13192821/s1, Table S1: Medicinal plants used to treat skin diseases and for cosmetic purposes in Norway; File S1: Questionnaire.
