# Supplementary material for: Medicinal Plants Used to Treat Skin Diseases and for Cosmetic Purposes in Norway
Source: Plants (Basel). 2024 Oct 9;13(19):2821. doi: 10.3390/plants13192821 (PMC11478695; doi:10.3390/plants13192821)
Supplement: Supplementary file 1 [file plants-13-02821-s001.zip › File S1.pdf]

File S1. Questionnaire

1. Respondent‘s gender:

- Female
- Male

2. Respondent‘s age:

3. Education, profession:

- Elementary
- Basic
- Secondary
- Higher
- Other

4. Residence:

5. How did you learn to treat with herbs?

- From parents, grandparents
- From neighbors, acquaintances
- From books, newspapers
- From radio, television, internet
- From a family doctor, pharmacist
- Other

6. Do you seek advice from your a pharmacist/doctor about using herbs to treat skin conditions?

- Pharmacist
- The doctor

- I do not apply (why)

7. Are natural conditions and the time of collection important to you for collecting herbs?

- Yes
- No

8. What conditions of nature and time are important for you when collecting herbs?

- Seasonality
- Time of the day
- Arrangement of celestial bodies, phases of the moon
- Weather conditions (sunny, rainy or foggy days)
- Other

9. Under what conditions and where do you store dried herbs?

10. What do you do with herbs that are no longer suitable for use?

- Burned
- Still consumed
- Bury in the ground
- You throw away

11. Do people appeal to you for help finding the herbal raw material they need?

- Yes
- No

12. Do you pass your experience of herbal treatment to other people?

- Yes
- No

13. What herbs do you collect?

14. What herbs do you grow yourself?

Structured part of the interview

15. For which skin diseases and which plants, their parts, preparation methods do you use?

16. For what cosmetic purposes and which plants, their parts, preparation methods do you use?

17. Compound recipes.
