# Supplementary material for: Medicinal Plants Used to Treat Skin Diseases and for Cosmetic Purposes in Norway
Source: Plants (Basel). 2024 Oct 9;13(19):2821. doi: 10.3390/plants13192821 (PMC11478695; doi:10.3390/plants13192821)
Supplement: Supplementary file 1 [file plants-13-02821-s001.zip › Table S1.pdf]

Table S1. Medicinal plants used to treat skin diseases and for cosmetic purposes in Norway

Family	Botanical name	Local plant name	Used for skin diseases - D, Used in cosmetics - C	Frequency of citations	Oral / external	Part used	Method of preparation	Usage	Therapeutic and cosmetic uses	EMA evaluation	Study area
Amaryllidaceae	*Allium cepa* L.	Løk	D	1	External	Bulb scales	Juice	Apply to skin	Wart removal		Bodø
Apiaceae	*Angelica archangelica* L.	Kvann	D	2	Oral, external	Root, whole plant, seed	Oil or alcohol extraction	Tincture, ointment, cream; Eat or drink as tea	For overloaded skin; bleeding wound		Oslo, Tjøme
Apiaceae	*Pimpinella anisum* L.	Anis	D	1	External	Seed	Oil extract	Bath	For overloaded skin	For symptomatic treatment of mild, spasmodic gastro- intestinal complaints including bloating and flatulence. Used as an expectorant in cough associated with cold	Oslo
Asphodelaceae	*Aloe vera* Mill.	Aloe vera	D	6	External	Leaves	Juice	Apply to skin	Wound healing, dry skin	EMA/807482/2016 Aloe vera folium; For short-term use in cases of occasional constipation	Bodø, Trondheim, Hamar, Tretten
Asteraceae	*Artemisia absinthium* L.	Malurt	D	1	External	Leaves	Decoction	In ointment	Insect bite, itchy skin	EMA/HMPC/13144/2020 temporary loss of appetite, mild dyspeptic/gastrointestinal disorders.	Hop

Asteraceae	*Artemisia vulgaris* L.	Burot	D	1	Oral	Whole plant	Cooked or raw	Eaten	For improvement of liver		Oslo
Asteraceae	*Arnica montana* L.	Arnica, solblom	D	2	External	Flower	Decoction	In ointment	Wounds healing	EMA/HMPC/198793/2012 or the relief of bruises, sprains and localised muscular pain.	Voss, Hop
Asteraceae	*Matricaria recutita* L.	Kamille	CD	8	Oral, external	Flower, leaves	Decoction, compress, oil extract	In ointment, oil infusion, cream, soap, tea	Wounds healing, bath Treatment of sensitive skin	EMA/490629/2015 Matricariae flos; Gastrointestinal disorders; Cough and cold; Mouth and throat disorders; Skin disorders & minor wounds	Arendal, Voss, Trondheim, Moss, Oslo, Færvik, Telemark
Asteraceae	*Arctium lappa* L.	Storborre	D	1	Oral	Root and seed	Alcohol or glycerin extract	Tea	Treatment of eczema and psoriasis	EMA/HMPC/246763/2009 used to increase the amount of urine to achieve flushing of the urinary tract as an adjuvant in minor urinary tract complaints; used in temporary loss of appetite; used in treatment of seborrhoeic skin conditions.	Oslo

Asteraceae	*Taraxacum officinale* (L.)	Løvetann	D	2	Oral, external	Flower, leaves	Decoction	Ointment, soap, tea, food	Eczema, psoriasis	EMA/HMPC/579636/2008 Taraxaci folium;<br><br>Urinary tract and genital disorders; Taraxaci radix cum herba; Gastrointestinal disorders; Loss of appetite; Urinary tract and gynecology disorders	Oslo, Moss
Asteraceae	*Centaurea cyanus* L.	Kornblomst	D	1	Oral, external	Flower, petals	Compress, oil extraction	Drink as tea, ointment	For relaxation, for dry lips and insect bites along with Calendula.	EMA/HMPC/228356/2012 Centaurii herba; Gastrointestinal disorders; Loss of appetite	Askøy
Asteraceae	*Calendula officinalis* L.	Ringblomst	CD	22	Oral, external	Flower, petals, stem, leaves, whole plant	Decoction Compress, oil extract	In ointment, cream, shampoo, soap, lip balm, tea	To treat dermatitis, eczema, and to reduce rashes. To soften hands. For lips. Athlete's foot. Acne. Minor wounds.	EMA/HMPC/228356/2012 Calendulae officinalis flos;<br>Skin disorders and minor wounds; Mouth and throat disorders	Bodø, Tjodalyng, Arendal, Tautra, Voss, Trondheim, Kristiansand, Brumunddal, Moss, Fredrikstad, Brattvåg, Hop, Hamar, Ringerike, Notodden, Tretten, Oslo, Risør, Færvik, Telemark,Tjøme, Askøy

Asteraceae	*Achilea millefolium* L.	Ryllik	CD	6	External	Flower, leaves	Decoction, oil extraction, vinegar extract	Bath, shampoo, soap, ointment	For bruises, wounds, chest tears. To stop bleeding. For acne and skin impurities.	EMA/HMPC/228356/2012 Millefolii flos; Loss of appetite; Gastrointestinal disorders; Skin disorders and minor wounds; Urinary tract and genital disorders	Arendal, Fredrikstad, Skaun, Oslo, Tjøme, Nesodden
Betulaceae	*Betula pubescens* L.	Bjørk	D	2	Oral, external	Leaves, buds, sap	Oil extract	Bath, tea, food	For unclean skin, psoriasis, eczema	EMA/HMPC/573241/2014 increase the amount of urine to achieve flushing of the urinary tract as an adjuvant in minor urinary complaints.	Oslo, Færvik
Brasicaceae	*Brassica oleraca* L.	Kål	D	1	External	Leaves	Soften leaves through rolling them out and applying them to skin	Apply to skin	For wound healing		Bodø
Boraginaceae	*Symphytum officinale* L.	Valurt	D	4	External	Roots, leaves, flower, whole plant	Compress Decoction, oil extract	Oil	Wound healing, for broken bones w/o internal bleeding	EMA/HMPC/333915/2015<br><br>Symphyti radix; Pain and inflammation	Fredrikstad, Skaun, Oslo, Nesodden
Caryophyllaceae	*Stellaria media* L.	Vassarve	D	2	Oral	Leaves	Extraction, oil extraction	Smoothie or tea, Shampoo, soap	For skin irritation		Oslo, Moss
Crassulaceae	*Rhodiola rosea* L.	Rosenrot	D	2	External, oral	Root	Decoction	Ointment, tea	Stress balance	EMA/HMPC/232091/2011 temporary relief of symptoms of stress, such as fatigue and sensation of weakness.	Hop, Oslo

Cupressaceae	*Juniperus communis* L.	Einer	CD	4	Oral, external	Needles, fruit, stem	Decoction; oil extract	Wound cream or ointment or wash. Berries are eaten.	For disinfection, for cleansing of skin, for cleansing of hair. For rheumatoid arthritis, sore muscles, psoriasis, pH imbalance.	EMA/HMPC/12402/2010 increase the amount of urine to achieve flushing of the urinary tract as an adjuvant in minor urinary tract complaints; symptomatic relief of digestive disorders such as dyspepsia and flatulence; adjuvant in the relief of minor muscular and articular pain.	Bodø, Færvik, Askøy, Nesodden
Equisetophyta	*Equisetum arvense* L.	Kjerringrokk, Åkersnelle	D	1		Leaves	Decoction		Wounds healing	EMA/HMPC/278091/2015 Equiseti herba; Urinary tract and genital disorders; For supportive treatment of superficial wounds.	Tretten
Fabaceae	*Trifolium pratense* L.	Rødkløver	D	3	External	Flower	Apply to skin, oil extract	In ointment	Psoriasis, facial scrub		Voss, Skaun, Hop
Grossulariaceae	*Ribes nigrum* L.	Solbær	D	1	External	Leaves	Decoction	In ointment	Eczema, psoriasis	EMA/HMPC/745353/2016 for the relief of minor articular pain; to increase the amount of urine to achieve flushing of the urinary tract as an adjuvant in minor urinary complaints.	Hop
Hypericaceae	*Hypericum perforatum* L.	Johannesurt	D	6	External	Aerial parts, whole plant, flower	Oil extract	In ointment, soap, oil infusion, oil	For sunburn. For sensitive and red skin.	EMA/HMPC/101304/2008<br><br>Hyperici herba; Mental stress and mood disorders; Skin disorders and minor wounds; Gastrointestinal disorders	Voss, Skaun, Hop, Ringerike, Telemark, Nesodden

Lamiaceae	*Prunella vulgaris* L.	Blåkoll	D	1	External	Flower	Extraction	Ointment	For tender varicose veins and hemorrhoids		Skaun
Lamiaceae	*Hyssopus officinalis* L.	Isop	D	1	External	Whole plant	Oil extract	Bath	Skin cleansing		Oslo
Lamiaceae	*Lavandula Spica* L.	Lavendel	CD	4	External	Flower, leaves	Decoction	In essential oil, ointment, cream, soap, shampoo	For skin irritation and itching	EMA/HMPC/734125/2010 for relief of mild symptoms of mental stress and exhaustion and to aid sleep.	Bodø, Trondheim, Kristiansand, Sunnfjord
Lamiaceae	*Mentha* spp.	Mynte	D	4	External	Leaves	Decoction, extraction	In ointment, cream, soap	Dry skin	EMA/HMPC/572705/2014 for the symptomatic relief of digestive disorders such as dyspepsia and flatulence; for use in the specified indication exclusively based upon long-standing use.	Bodø, Oslo, Tjøme, Sunnfjord
Lamiaceae	*Origanum vulgare* L.	Oregano	C	1	External	Leaves	Oil extraction	Oil	Unclean skin		Tjøme
Lamiaceae	*Salvia officinalis* L.	Salvie	D	1	External	Leaves	Decoction	Bath	For wrinkly skin	EMA/HMPC/277152/2015 for relief of mild dyspeptic complaints such as heartburn and bloating; for relief of excessive sweating; for relief of inflammations in the mouth or the throat; for relief of minor skin inflammations.	Oslo

Lamiaceae	*Salvia rosmarinus* L.	Rosmarin	CD	3	External	Leaves	Extraction	Bath, hair oil	For hair, mild psoriasis and eczema on scalp	EMA/HMPC/513940/2021 symptomatic relief of dyspepsia and mild spasmodic disorders of the gastrointestinal tract; for the relief of minor muscular and articular pain and in minor peripheral circulatory disorders.	Oslo, Tjøme, Sunnfjord
Lamiaceae	*Thymus vulgaris* L.	Timian	D	1	External	Leaves	Extract	Soap	For cleansing of skin	EMA/HMPC/342332/2013 used in productive cough associated with cold.	Notodden
Malvaceae	*Malva sylvestris* L.	Mauretansk kattost	D	1	External	Flower	Extraction	Ointment	For sensitive skin	EMA/HMPC/749511/2016 used as a demulcent preparation for the symptomatic treatment of oral or pharyngeal irritation and associated dry cough; used as a demulcent preparation for the symptomatic relief of mild gastrointestinal discomfort.	Moss
Myrtaceae	*Eucalyptus globulus* Labill.	Eukalyptus	D	1	External	Leaves	Oil extraction	Oil	For dry skin	EMA/HMPC/307781/2012 for relief of cough associated with cold; symptomatic relief of localised muscle pain.	Trondheim
Pinaceae	*Picea abies* L.	Gran	D	1	External	Sap, shoots	Decoction, oil extraction	Ointment	for muscle pain and rheumatism		Oslo

Pinaceae	*Pinus sylvestris* L.	Furu	D	1	External	Sap, needles	Decoction, oil extraction	Ointment	For skin irritation		Notodden
Plantaginaceae	*Plantago major* L.	Groblad	D	12	External, oral	Leaves	Decoction, extract	In ointment, cream, soap, shampoo	Wounds healing. To stop bleeding. For eczema and psoriasis.		Bodø, Arendal, Moss, Skaun, Hamar, Notodden, Tretten, Oslo, Tjøme, Nesodden
Rosaceae	*Rubus idaeus* L.	Bringebær	D	1	External	Leaves	Decoction	In ointment	For wound healing	EMA/HMPC/44211/2012 for the symptomatic relief of minor spasm associated with menstrual periods; for the symptomatic treatment of mild inflammation in the mouth or throat; for the symptomatic treatment of mild diarrhoea.	Hop
Rosaceae	*Alchemilla vulgaris* L.	Marikåpe	D	3	External, oral	Leaves	Extract, Tea, apply directly to skin (beestings)	Cream, ointment, tea	Wound healing, abrasions, treatment of bee stings, lymphatic system		Skaun, Ringerike, Oslo
Rosaceae	*Rosa rugosa* L.	Rose	D	2	External, oral	Flower, root	Extraction	Ointment, cream, juice, tea	Improves circulation, stress management. As anti-aging agent and moisturizer.		Trondheim, Brumunddal

Rosaceae	*Rosa rugosa* L.	Nype	D	1	External, oral	Fruit	Extract, oil extract	Drink as juice, used in ointment	For hair treatment, as universal ointment, skin cleansing		Arendal
Rubiaceae	*Galium aparine* L.	Klengemaure	D	1	Oral	Whole plant	Tea, smoothie	Drink as tea or smoothie	Treatment of eczema and wound healing		Oslo
Urticaceae	*Urtica dioica* L.	Brennesle	CD	7	Oral, external	Aerial parts	Decoction, extract	Ointment, cream, shampoo	For dry skin, wound healing, dry hair, to stop bleeding and nosebleeds.	EMA/HMPC/461160/2008 for the relief of lower urinary tract symptoms related to benign prostatic hyperplasia after serious conditions have been excluded by a medical doctor.	Bodø, Voss, Moss, Hop, Notodden, Tretten, Færvik
